# Presence of antibodies against cyclic citrullinated peptides in patients with 'rhupus': a cross-sectional study

**DOI:** 10.1186/ar2036

**Published:** 2006-08-25

**Authors:** Luis M Amezcua-Guerra, Rashidi Springall, Ricardo Marquez-Velasco, Lorena Gómez-García, Angélica Vargas, Rafael Bojalil

**Affiliations:** 1 Department of Immunology, Instituto Nacional de Cardiología Ignacio Chávez, Juan Badiano 1, Sección XVI, Tlalpan 14080, Mexico City, Mexico; 2 Department of Rheumatology, Instituto Nacional de Cardiología Ignacio Chávez, Juan Badiano 1, Sección XVI, Tlalpan 14080, Mexico City, Mexico; 3 Department of Health Care, Universidad Autónoma Metropolitana Xochimilco, Calzada del Hueso 1100, Villa Quietud, Coyoacán 04960, Mexico City, Mexico

## Abstract

'Rhupus' is a rare condition sharing features of rheumatoid arthritis (RA) and systemic lupus erythematosus (SLE). If rhupus is a distinctive entity, an overlap between RA and SLE or a subset of SLE is currently debated. This study was performed to explore the prevalence of antibodies against cyclic citrullinated peptides (anti-CCP antibodies) in rhupus. Patients meeting American College of Rheumatology criteria for RA, SLE, or both were included. Clinical and radiographic features were recorded and sera were searched for anti-CCP antibodies, rheumatoid factor, antinuclear antibodies, anti-extractable nuclear antigens, and antibodies against double-stranded DNA (anti-dsDNA antibodies). Seven patients for each group were included. Clinical and serological features for RA or SLE were similar between rhupus and RA patients, and between rhupus and SLE patients, respectively. Values for anti-CCP antibodies obtained were significantly (*p *< 0.05) higher in RA (6/7) and rhupus (4/7) than in SLE patients (0/7) and healthy subjects (0/7). Our data support the possibility that rhupus is an overlap between RA and SLE, because highly specific autoantibodies for RA (anti-CCP) and for SLE (anti-dsDNA and anti-Sm) are detected in coexistence.

## Introduction

The clinical coexistence of rheumatoid arthritis (RA) and systemic lupus erythematosus (SLE) was first described in 1969 by Kantor and was termed 'rhupus syndrome' by Schur (both cited in [1]). Since then, fewer than 100 cases of rhupus have been published [1-3]. In an epidemiological study including about 7,000 new patients, the prevalence of RA was 15% and for SLE it was 8.9%. The expected coincidence of RA and SLE by chance would therefore be 1.2%. However, the observed prevalence of rhupus was 0.09%, less than one-tenth of that expected [1].

Previous reports have shown that the patients with rhupus display an array of autoantibodies including anti-double-stranded DNA (anti-dsDNA), anti-Sm (both highly specific for SLE), anti-SSA, anti-SSB, anti-ribonucleoprotein, antinuclear antibodies (ANA), anti-cardiolipins, and rheumatoid factor (RF) [1,2]. However, no study has yet been performed to investigate the presence of antibodies against cyclic citrullinated peptides (anti-CCP antibodies), which have a specificity for RA of 96 to 98% (for second-generation assays (anti-CCP2)) [4,5]. Recent data have confirmed that these antibodies are rarely if ever present in other autoimmune diseases such as SLE, Sjögren's syndrome (SS), scleroderma and myositis [6]. Nowadays, it is a matter of debate whether rhupus is a clinically and immunologically distinctive entity [2], a true overlap between SLE and RA [7], or a subgroup of patients with lupus [8].

This descriptive, cross-sectional study was performed to investigate the frequency of anti-CCP antibodies in a cohort of patients with rhupus.

## Materials and methods

We included all patients fulfilling American College of Rheumatology (ACR) classification criteria for both RA [9] and SLE [10] who belonged to our cohorts of patients with RA and with SLE. Comparisons were made with age- and gender-matched patients with RA and with SLE, and healthy subjects. The study was approved by the local ethics committee, and informed consent was obtained. Serum samples were obtained and stored at -75°C until use. Sera were analyzed for anti-CCP2 antibodies by ELISA (Inova Diagnostics, San Diego, CA, USA) with a cutoff value of 60 U/ml. Fine antinuclear reactivities (ELISA; Inova Diagnostics), RF (nephelometry), ANA (indirect immunofluorescence on HEp-2 slides), and anti-dsDNA (indirect immunofluorescence on *Crithidia luciliae *substrate) antibodies were also determined. Except for healthy individuals, standard radiographs of hands were available. For statistical analysis, ANOVA and the Mann–Whitney *U *test were performed as appropriate with GraphPad Prism 4.0 software (GraphPad Inc, San Diego, CA, USA).

## Results

Seven female patients with a median age of 44 years (range 25 to 64) met our inclusion criteria. The major clinical and laboratory findings are presented in Table 1. Healthy individuals and all patients belonged to cohorts from the same ethnic group (Hispanic mestizo). No differences in demographic data were found between groups.

Mean ACR criteria for SLE were 5.57 (range 4 to 8) in the SLE group, and 5.57 (4 to 8) in the rhupus group. In the same way, mean ACR criteria for RA were 6 (4 to 7) in the RA group, and 5.14 (4 to 6) for the patients with rhupus. In all patients with rhupus, RA was presented as the initial disease, as has been described previously [2]. In accordance with another report, in two patients the disease started during their childhood as juvenile chronic arthritis [1].

**Figure 1:**
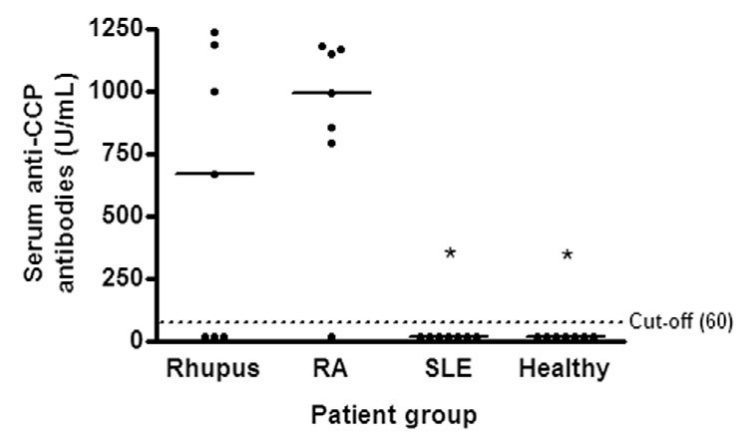
Analysis of serum anti-CCP antibodies. Patients with rhupus and those with rheumatoid arthritis (RA) had significantly higher serum antibodies against cyclic citrullinated peptides (anti-CCP antibodies) than patients with systemic lupus erythematosus (SLE) and healthy subjects (*p < 0.05). Results are expressed in U/ml for each patient. Horizontal lines indicate the median value for anti-CCP antibodies in each group. The dotted line represents the cutoff value for positivity (60 U/ml).

Anti-CCP antibodies were found in four of seven (57%) patients with rhupus, and in six of seven (86%) patients with RA, whereas neither patients with SLE nor healthy individuals showed reactivity. When the concentrations in each group were compared, a statistical significant difference between groups was found (ANOVA, *p *< 0.05). The mean concentration of anti-CCP antibodies was 584 U/ml (range 0 to 1,237) in patients with rhupus (Figure 1), 875 U/ml (0 to 1,178) in the RA group (not significant compared with rhupus), 1 U/ml (0 to 10) for SLE individuals (*p *< 0.05 compared with rhupus), and 0 U/ml (0 to 2) for healthy controls (*p *< 0.05 compared with rhupus). Two of three patients with rhupus who were negative for anti-CCP antibodies were also negative for anti-dsDNA antibodies, RF and anti-extractable nuclear antigen antibodies, although both patients met RA and SLE classification criteria, including ANA and erosive arthritis.

Differences in ANA, anti-dsDNA and anti-extractable nuclear antigen autoantibodies between patients with rhupus and those with SLE were not found. We also found no difference in the prevalence of RF between patients with rhupus and those with RA. Surprisingly, one healthy subject was positive for RF, ANA and anti-SSA antibodies, although she was asymptomatic and no features of any disease were found.

## Discussion

The close association between different type II human leukocyte antigen (HLA) molecules and the risk of RA is well established. These major histocompatibility complex (MHC) class II molecules share the same amino acid sequence (QKRAA or QRRAA) in positions 69 to 74 of the β-chain, namely the 'shared epitope'. Recent works have demonstrated that this 'shared epitope' preferentially binds peptides containing the non-standard amino acid citrulline (deiminated arginine) [11]. In addition, an abnormally increased function of the enzyme peptidylarginine deiminase 4 (PAD4; responsible for the deimination of arginine) and an elevated anti-CCP autoantibody production in patients with RA have been demonstrated [12]. These facts have built the first bridge between cellular and humoral autoimmunity in a major rheumatic disease, supporting a pathogenetic role for an abnormal metabolism of citrulline in the development of RA [13,14].

Patients with SLE are often part of the control group when determining the specificity of anti-CCP antibodies for RA [15], although some studies have been performed specifically on patients with SLE. These studies contribute some clues to the role of anti-CCP antibodies in rhupus. Mediwake and colleagues [16], in a study exploring the predictive value of anti-CCP antibodies to distinguish erosive arthritis in SLE, found ten patients (out of 231) with erosive arthritis, two of whom had anti-CCP antibodies. In concord with this, Hoffman and colleagues [15] demonstrate that three patients with erosive arthritis, included in a cohort of 235 patients with SLE, were positive for anti-CCP antibodies. These authors suggest that the presence of anti-CCP antibodies can predispose for a chronic RA-like arthritis in patients with SLE. Additionally Weissman and colleagues [17] demonstrated that patients with SLE can display radiographic abnormalities similar to those of RA, although the presence of marginal erosions is a rare finding.

In the present study we demonstrate that the patients with rhupus show a very similar arthritis pattern (including erosive disease) and similar autoantibody production (RF and anti-CCP antibodies) to those in patients with RA. In addition, patients with rhupus display a clinical and serological profile indistinguishable from patients with SLE. Moreover, the presence of other coexistent autoimmune diseases was similar in all groups of patients (two patients with rhupus, three patients with RA, and three patients with SLE also had SS).

We found high titers of anti-CCP antibodies in four of seven (57%) patients with rhupus, a frequency similar to that reported for RA [4]. This finding, together with the clinical similarity, supports the contention that rhupus belongs to the RA spectrum. The high prevalence of anti-CCP antibodies in RA found in our study could be explained by a selection bias because only patients with RA with an aggressive disease (namely erosive arthritis and RF^+^) were included. In contrast, the mean ACR criterion for SLE was similar between patients with rhupus and those with SLE, including the 'robust' features of SLE such as renal and neurological involvement, and anti-dsDNA and anti-Sm antibodies. These clinical and serological features shared between patients with rhupus and those with SLE also place rhupus in the SLE spectrum.

Titration of anti-CCP antibodies in the rhupus group clearly shows a bimodal distribution, suggesting the existence of two different subpopulations. Because of the small number of patients, we are unable to define the differential features underlying each subset. However, two of three patients negative for anti-CCP antibodies were also negative for both RF and anti-dsDNA antibodies.

## Conclusion

On the basis of the presence of shared clinical features of RA (mainly erosive arthritis) and SLE (including renal and neurological involvement) along with the presence of anti-dsDNA and anti-CCP autoantibodies in our patients with rhupus, our findings strongly support the contention that rhupus is a true overlap between RA and SLE, not merely a part of the clinical spectrum of the articular involvement seen in SLE. Moreover, on the basis of the mean ACR criteria for both diseases, we have confirmed that patients with rhupus have more RA-associated and less SLE-associated damage, an issue that has been suggested previously [2].

To our knowledge, this is the first report exploring the prevalence of anti-CCP antibodies specifically in patients with rhupus. More studies are needed to expand the pathogenetic knowledge of this overlap syndrome.

## Abbreviations

ANA = antinuclear antibodies; anti-CCP antibodies = antibodies against cyclic citrullinated peptides; anti-dsDNA antibodies = antibodies against double-stranded DNA; ELISA = enzyme-linked immunosorbent assay; RA = rheumatoid arthritis; RF = rheumatoid factor; SLE = systemic lupus erythematosus; SS = Sjögren's syndrome.

## Competing interests

The authors declare that they have no competing interests.

## Authors' contributions

LA-G participated in the conception and design of the experiments, in the acquisition, analysis and interpretation of data, and was involved in drafting the manuscript. RS performed the immunoassays. RM-V participated in the analysis and interpretation of data and performed the statistical analysis. LG-G participated in the analysis and interpretation of data. AV participated in the recruitment of patients and the acquisition of data. RB participated in the interpretation of data, revising the manuscript for intellectual content and giving the final approval of the version to be published. All authors read and approved the final manuscript.

## Figures and Tables

**Table 1:** Clinical and serological features of patients by study group

Characteristics	Rhupus	RA	SLE	Controls
Number of patients	7	7	7	7
Median age, years (range)	44 (25–64)	46 (31–61)	41 (24–66)	41 (28–63)
Median age of onset, years (range)	26 (11–61)	40 (27–45)	39 (18–52)	-
SLE criteria, *n *(percentage)				
Malar rash	5 (71)	-	7 (100)	-
Discoid lupus	1 (14)	-	1 (14)	-
Photosensitivity	4 (57)	-	5 (71)	-
Oral ulcers	5 (71)	-	5 (71)	-
Serositis	3 (43)	-	0 (0)	-
Renal	2 (29)	-	2 (29)	-
Neurological	1 (14)	-	1 (14)	-
Hematological	7 (100)	-	6 (86)	-
Immunological	4 (57)	-	5 (71)	-
ANA	7 (100)	-	7 (100)	-
RA criteria, *n *(percentage)				
Morning stiffness	3 (43)	5 (71)	-	-
Arthritis in more than three areas	7 (100)	7 (100)	-	-
Arthritis of hands	7 (100)	7 (100)	-	-
Symmetric arthritis	7 (100)	7 (100)	-	-
Rheumatoid nodules	0 (0)	3 (43)	-	-
RF	5 (71)	6 (86)	-	-
X-ray changes	7 (100)	7 (100)	-	-
Serology, *n *(percentage)				
Anti-CCP	4 (57)	6 (86)	0 (0)	0 (0)
ANA	7 (100)	0 (0)	7 (100)	2 (29)
Anti-dsDNA	4 (57)	0 (0)	4 (57)	0 (0)
Anti-SSA	4 (57)	0 (0)	3 (43)	1 (14)
Anti-SSB	1 (14)	0 (0)	1 (14)	0 (0)
Anti-Sm	1 (14)	0 (0)	2 (29)	0 (0)
Anti-RNP	1 (14)	0 (0)	2 (29)	0 (0)
RF	5 (71)	6 (86)	2 (29)	1 (14)

ANA, antinuclear antibodies; anti-CCP, antibodies against cyclic citrullinated peptides; anti-dsDNA, antibodies against double-stranded DNA; anti-RNP, antibodies against ribonucleoprotein; RA, rheumatoid arthritis; RF, rheumatoid factor; SLE, systemic lupus erythematosus; SS, Sjögren's syndrome.
